# Isoform sequencing provides insight into natural genetic diversity in maize

**DOI:** 10.1111/pbi.13063

**Published:** 2019-06-14

**Authors:** Yong Zhou, Zhixuan Zhao, Zhiyong Zhang, Miaomiao Fu, Yongrui Wu, Wenqin Wang

**Affiliations:** ^1^ Department of Plant Sciences School of Agriculture and Biology Shanghai Jiao Tong University Shanghai China; ^2^ National Key Laboratory of Plant Molecular Genetics CAS Center for Excellence in Molecular Plant Sciences Institute of Plant Physiology & Ecology, Shanghai Institutes for Biological Sciences Chinese Academy of Sciences Shanghai China; ^3^ University of the Chinese Academy of Sciences Beijing China

**Keywords:** isoform sequencing, W64A, endosperm texture, molecular marker, genetic diversity

W64A, as a member of non‐stiff stalk maize, has been used to develop current corn in plant breeding, and serving as one of broadest parent lines for the commercial hybrid seed production (Huffman, 1984). The inbred had the characteristics of early flowering, average plant and ear height at its maturity, very strong roots and good stalks (Runge *et al*., 2004). In addition, W64A serves as an invaluable germplasm to study gene functions especially in the field of corn nutrition and endosperm texture given its good vitreousness and hardness (Figure 1a). However, little is known about the background of genetic and genomic information for W64A. With the advent of the revolutionary technology of PacBio long‐read sequencing, we can simultaneously obtain a large amount of full‐length cDNA up to 20 kb (An *et al*., 2018).

**Figure 1:**
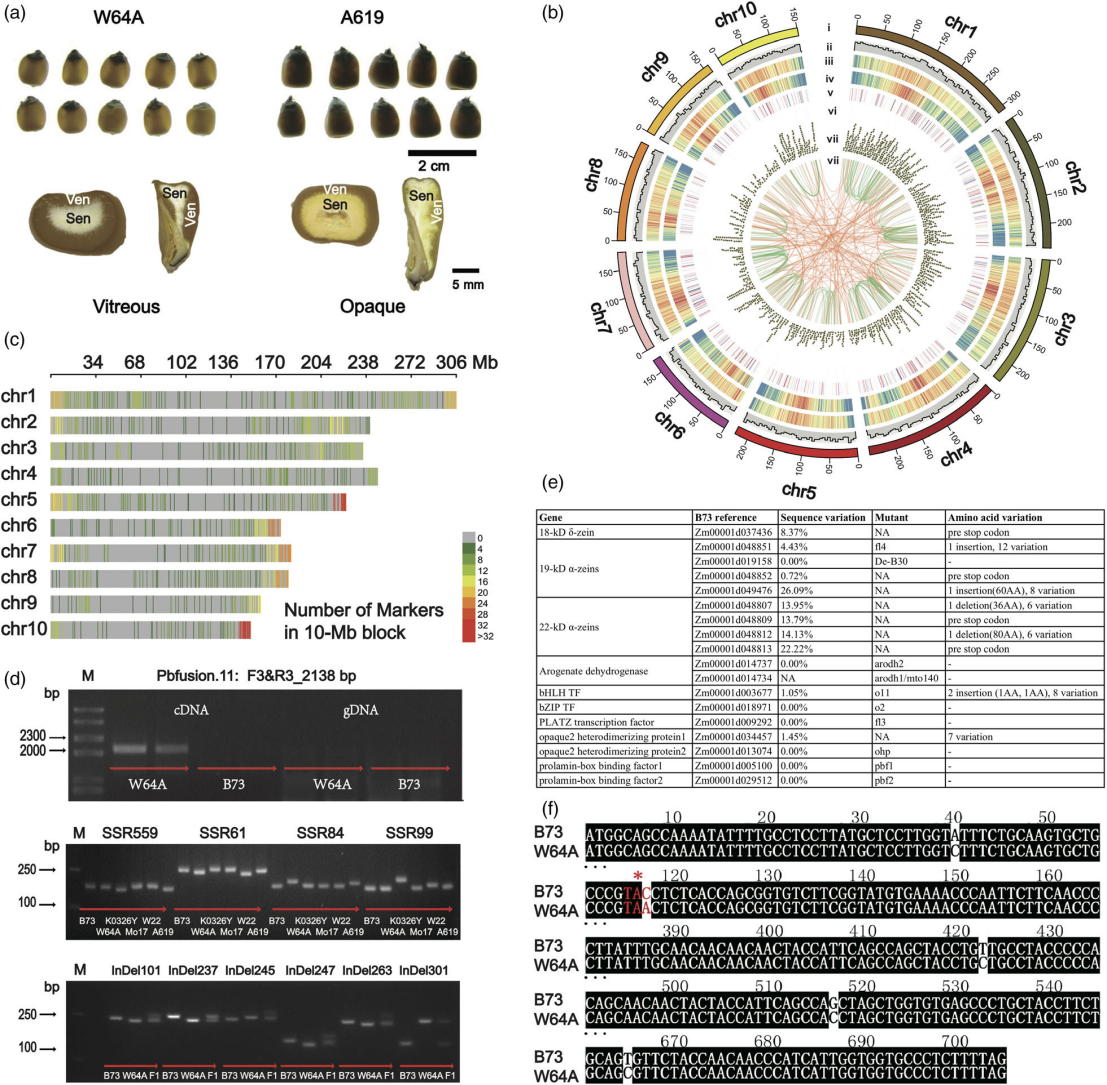
Isoform sequencing of W64A provides insights into transcriptome complexity in maize. (a) The endosperm texture of W64A (vitreous) and A619 (opaque). ‘Sen’ means starchy endosperm. ‘Ven’ indicates vitreous endosperm. (b) Transcript distribution in CIRCOS visualization. (i) Ten chromosomes of B73 maize genome. (ii) The number of novel loci in a 5‐Mb window for W64A. (iii) and (iv) The gene density in a 1‐Mb window for W64A and B73. (v) and (vi) The number of AS events in W64A and B73. (vii) The lncRNA density of W64A in a 1‐Mb window. (viii) The linkage of fusion transcripts at intra‐chromosome (green) and inter‐chromosome (orange) in W64A. (c) The SSR and InDel marker distribution in a 10‐Mb block. (d) The validations for fusion genes (top), SSRs (middle) and InDels (bottom). “M” is a DNA ladder. (e) The sequence diversity of genes associated with endosperm texture (Zhang *et al*., 2018) compared W64A with B73. (f) An example of 19‐kDa zein alignment that the second SNP creates a pre‐stop codon in W64A.

To accelerate and improve the germplasm breeding, we sequenced and characterized the full‐length transcriptomes of *Zea mays* W64A using the PacBio long‐read sequencing technology. High‐quality RNA was prepared from the W64A endosperm at 16 days after pollination (DAP). Three libraries including 1–2 kb, 2–3 kb, and 3–6 kb were constructed using BluePippin size selection system. The libraries were sequenced on the PacBio RS II using the P6‐C4 chemistry with 16 SMRT cells, yielding 1 057 799 polymerase reads with the average length of 17 868 bp. There were 494 798 full‐length non‐chimeric (FLNC) reads which contained 5′ primer, 3′ primer and the poly (A) tail. The length distributions of read of inserts were expected as each library established, with an average of 1755, 2889, and 3766 bp, respectively. The number of full‐length non‐chimeric reads was very close to that of full‐length reads, indicating the low number of artificial concatemers and a high quality of the SMRT library preparation. The full‐length non‐chimeric reads were further clustered and a total of 166 693 unique isoforms were achieved that were subjected to the downstream analysis. The PacBio sequences were deposited in NCBI Sequence Read Archive with the accession number of SRP155967 and the BioSample accession of SAMN09747820 under BioProject ID of PRJNA483809.

We mapped the 166 693 full‐length isoform sequences to the maize reference (RefGen_v4.0) (Jiao *et al*., 2017). It was found that 166 103 (99.65%) isoforms were mapped to the reference. Among them, 144 721 isoforms (86.8%) were mapped to gene regions, amounting to 21 672 known gene loci. Still, a number of 21 382 isoforms were mapped to the B73 intergenic regions, predicted to be 3399 novel gene loci by blastX against NCBI nr database (Figure 1b). There were 75 711 novel isoforms were located in the known gene loci mainly due to alternative splicing (AS). Each known gene locus contained 6.68 isoforms, whereas each novel locus had 1.49 isoforms. The low number of isoforms for novel gene loci may be one of reasons why it was hard to detect in previous studies of RNA sequencing. A similar pattern of AS models was found in W64A and B73 (Wang *et al*., 2016) (Figure 1b), where alternative 3′ (86 244, 37.50%) and 5′ (84 105, 36.60%) splicing events were more than those of exon skipping (25 828, 11.20%) and intron retention (33 725, 14.70%). After filtering potential ORFs and coding sequences, the pipeline of PLEK predicted a number of 1167 known long non‐coding transcripts (lncRNAs) and 879 novel lncRNAs (Figure 1b). There were 45%, 21%, and 34% of lncRNAs mapped to the intronic, exonic, and intergenic region, respectively. A number of 1527 fusion transcripts were defined with an average length of 1528 bp with the optimized and stringent parameters in terms of the sufficient aligned length and the spanning distance (Figure 1b). More intra‐chromosome fusion events (1301, 85.20%) were found than the inter‐chromosome fusion transcripts (226, 14.80%). Ten candidate fusion transcripts were randomly chosen to run the validation. Eight events were present in their fusion transcripts, whereas two had negative results that could be generated from chimeric reads in PacBio sequencing (Figure 1d top).

A total of 1051 Simple sequence repeats (SSRs) and 243 insertions/deletions (InDels) (Figure 1c) were identified with an average frequency of 5.3 SSRs/Mb and 1.2 InDel/10Mb, respectively. We set the strict parameters that only allowed more than 5‐bp InDel could be retrieved after comparing ROIs of W64A to the reference genome sequences. More than 20 bp of the repeat length were defined as a potential SSRs. The e‐PCR primers flanking the molecular markers were designed according to the maize reference of B73. We randomly picked 100 candidate SSRs and validated their polymorphisms by agarose gel electrophoresis, and found that 63% of the SSRs presented their diversity among six maize inbred lines (B73, W64A, Mo17, K0326Y, W22, and A619) (Figure 1d middle). All InDel markers were confirmed by PCR and agarose gel electrophoresis, leading to 123 (58.8%) being effective (Figure 1d bottom).

To further provide the reference sequences and to understand the formation of the vitreous endosperm in W64A, we surveyed the genes associated with endosperm texture based on previous studies (Zhang *et al*., 2018). We found similar copy numbers of opaque‐associated genes, including zein, non‐zein, enzyme and transcription factor genes, where they presented an average sequence diversity of 3.92% with a highest number of 26.9% (Figure 1e). The four‐nucleotide deletion of ‘TTAT’ in 18‐kDa created a reading frame shift, resulting in a pre‐stop codon in W64A. The 18‐kDa protein was validated to be missing in W64A but present in B73 by using an SDS‐PAGE (Wu *et al*., 2009). Certain genes showed more than 20% sequence variation when comparing W64A and B73 including members of 19‐kDa and 22‐kDa. The second SNP of ‘C’ to ‘A’ transition in a zein member of 19‐kDa (Zm00001d048852) caused a pre‐stop codon (Figure 1f). Two InDels were identified in the coding region of *O1* gene, leading to a seven amino‐acids deletion and a twenty‐four amino‐acid insertion compared with B73. It was reported that the O1 protein was involved in protein body biogenesis by affecting the RER motility so that the mutant produced opaque kernels (Hunter *et al*., 2002).

Here, we generated 166 693 high‐quality isoforms with an average length of 2715 bp for W64A developing endosperm using the PacBio long‐read sequencing technology. They were annotated 21 672 known gene loci, as well as 3399 novel gene loci, covering 63% of the filtered gene set from the reference of B73. A number of 1051 SSRs and 243 InDels were generated in comparison to the B73 genome, which could be easily visualized by gel electrophoresis without any further cloning and enzyme digestion. The transcriptome dynamics was confirmed by high levels of sequence divergence at genes associated with endosperm texture, suggesting that the genetic variation may affect the phenotypic differences. The isoform sequencing of W64A not only provided new insights into transcriptome complexity in maize endosperm, but also supply a resource of molecular markers for the quantitative trait locus (QTL) mapping and further genetic breeding.

## Conflict of interest

The authors declare no conflict of interest.
